# Towards Co-Design in Delivering Assistive Technology Interventions: Reconsidering Roles for Consumers, Allied Health Practitioners, and the Support Workforce

**DOI:** 10.3390/ijerph192114408

**Published:** 2022-11-03

**Authors:** Natasha Layton, Jackie O’Connor, Amy Fitzpatrick, Sharon Carey

**Affiliations:** 1 Rehabilitation, Ageing and Independent Living (RAIL) Research Centre, Monash University, Clayton, Melbourne, VIC 3800, Australia; 2 Australian Rehabilitation and Assistive Technology Association (ARATA), Frankston, VIC 3199, Australia; 3 Nutrition and Dietetics Department, Royal Prince Alfred Hospital, Camperdown, Sydney, NSW 2050, Australia

**Keywords:** allied health, assistive technology, policy, person centered practice, co-design, workforce, occupational therapy, prosthetics, orthotics, speech pathology, dietetics, consumer directed care

## Abstract

A complexity of factors, from health and technology innovations to policy redesign to achieve consumer-directed care, are impacting traditional roles for Australian allied health practitioners (AHPs). This pilot study considers roles for AHPs in relation to assistive technology (AT) interventions. Articulating ‘who does what’ may serve a number of purposes including de-professionalization of the discourse; better utilization of support networks and workforces; and alignment with contemporary policy. Yet, a suitable framework to assist with collaborative AT implementation between relevant stakeholders was not identified within the existing literature. This research aimed to develop and pilot an AT collaboration tool which enables AHPs, consumers, their support networks and the support workforce, to navigate policy redesign toward ethical consumer-directed implementation of AT interventions. An AT collaboration tool was developed based upon practice-based knowledge, relevant regulatory and practice evidence and identifies relevant stakeholders, AT service steps and roles, and quality indicators to support competent practice. The tool was piloted in four separate and diverse practice analyses of AT interventions (custom prosthetics, home enteral nutrition, communication devices, and vehicle modifications) considering four allied health professions (prosthetics and orthotics, dietetics, speech pathology, occupational therapy). Pilot testing of the tool supports the feasibility of re-framing AT provision using competency-based and risk-informed approaches and enabling more inclusive roles for consumers and the support workforce. Further testing of the tool is indicated, followed by strategic actions for uptake by individuals, professions and policymakers. The AT collaboration tool has potential to enable AHPs to fulfil ethical obligations for consumer-centered practice, and to facilitate consumer choice, both in Australia and internationally.

## 1. Introduction

### 1.1. Supercomplex Environments and Allied Health Professions

Allied health practitioners (AHP) operate within ‘supercomplex’ health and social care systems [1,2]. Some of the overarching factors at play include the paradigm shifts from deficit-based models to a bio-psycho-social approach [3,4]; developments in rehabilitation to focus on functioning, participation and enablement [5,6]; and the development of personalized or ‘n of 1’ medicine [7]. The evidence base of allied health professions is also dynamic and the evolution of individual professions over time can be seen in profession-specific journals and through the competency standards of various professional associations. Allied health practice has long adhered to evidence-based practice principles [8] and is also shaped by imperatives to work with service users [9] and to integrate experience-based co-design into services [10,11].

Policy redesign towards consumer-directed care and individualized funding also impact allied health practice. Many countries are reconstructing disability and aged care policy to person-centered care, which places consumers and their individual goals in the center of service systems [12], including Australia [13,14]. This represents a substantial change to the previous service contexts in which AHPs worked. Person-centered care is consistent with integrated care principles [15] and promises to strengthen the consumer voice during health interventions [16,17]. An absence of choice and control for consumers and their families under the previous systems is acknowledged [18] and a realignment of ‘power‘ is occurring in the form of individualized plans and budgets alongside consumer discretion over the services and therapies (including AT) upon which these budgets can be spent [19]. In essence, consumers must select their own allied health practitioner and nominate their therapy of choice within some broad commitments to evidence-based approaches [20].

#### Assistive Technology—A Key Intervention for Many Allied Health Professions

AT is an umbrella term for assistive products and related services. Assistive products refer to specifically made or mainstream devices or software which can maintain or improve people’s ability to function and live independently.

Allied health professions in Australia includes multiple professions. AT is an intervention used by a subset of professions including occupational therapy, speech and language pathology, podiatry, dietetics, physiotherapy and prosthetics and orthotics. Professions outside allied health, such as nursing and rehabilitation engineers, may also have some aspects of AT within their scope of practice.

Australian and international standards classify over 650 types of assistive product categories with many thousands of individual products. There are twelve broad classes of assistive products related to AHPs for whom they are within scope. These can be seen in Appendix A [21]. There are one or more AHP roles listed for each product class. Roles were matched based upon prescribing conventions in Australia, that is, the AHPs nominated as prescribers in equipment funding schemes as well as the authors’ experiences.

Assistive products are only effective if they are tailored to the user, user goals, and the environments of use. Forty years of AT research demonstrates this tailoring requires AT services or ‘wraparound services’ including screening and assessment, product selection, product fitting and adjustment, user training, and follow up for review and maintenance and are summarized in the World Health Organization Position Paper on AT provision [22]. These elements have often been provided by AHPs, as described in Appendix A, or through the skilling of alternative workforces such as community-based rehabilitation workers in low- and middle-income countries [23,24,25].

### 1.2. The Australian Context

Over the last decade, Australian national disability and aged care acts and policies have been rewritten to reflect the philosophies of consumer directed care alongside market economics: referred to hereafter as the National Disability Insurance Scheme (NDIS) and My Aged Care (MAC) [26,27]. The repositioning of the service recipient as a consumer of services has many consequences. This repositioning may place significant demands upon consumers to manage this marketized approach to purchasing the services necessary for AT interventions [28]. Consumers are expected to have clear ideas of their goals and understanding of how and what AT may contribute to achieving them; to make choices about which practitioner they wish to assist them in provision of AT; and whether they require a practitioner at all to mediate their AT choices [29,30]. Much AT provision, previously managed by an AHP, can now be initiated by consumers themselves, with minimal sign off from funder delegates such as package brokers (My Aged Care) or planning or local area co-ordination staff (NDIS).

If consumers are to be afforded the opportunity for self-directed care whilst continuing to access the wraparound services required for successful AT implementation, all stakeholders need to understand who is performing which tasks (‘who does what’). Guidance tools would need to align with contemporary policy and practitioner and ethical obligations, and to be inclusive of consumers, their support networks, alternative workforces and AHPs. Such guidance or frameworks were not identified within the existing peer reviewed or practice support (grey) literature.

This research aimed to develop and pilot an AT collaboration tool which enables AHPs, consumers, their support networks and alternative workforces, to navigate policy redesign toward ethical consumer-directed implementation of AT interventions.

## 2. Method

**Figure 1:**
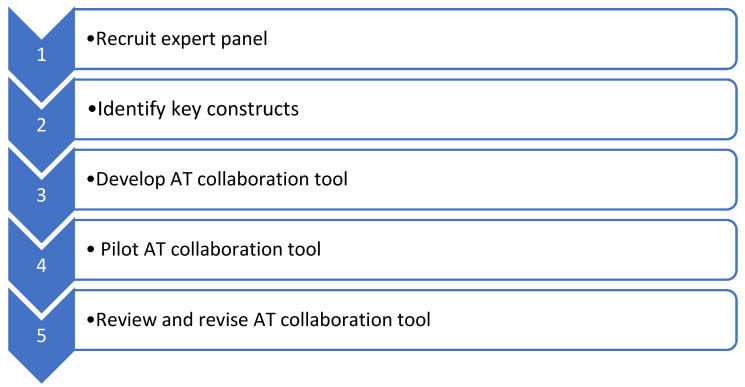
Method of iterative development and testing.

An iterative development and case study test, review and revise approach was used to (a) develop and (b) pilot an AT collaboration tool. Figure 1 lists these steps which are described below.

### 2.1. Step 1: Recruit Expert Panel

An intersectional lens was used to identify practitioner participants for the development and piloting of the AT collaboration tool. Involvement was sought across the key professions listed in Appendix A. Key characteristics to consider capability included: substantive practice knowledge; experience in at least one of policy design and implementation; and/or use of AT as a consumer. A minimum of four disciplines were to be represented.

Participants were invited to join the panel to develop and pilot the tool through dialogue with allied health practitioner associations and communities of practice such as ARATA (Available online: www.arata.org.au (accessed on 1 March 2021)). Due to the exploratory and iterative nature of the study, only one expert from each discipline was recruited.

### 2.2. Step 2: Identify Key Constructs

#### Existing Frameworks Relevant to Co-Design of AT Interventions

The literature was identified based on the collective expertise and knowledge of the field of our team members, and through literature search using Google Scholar to locate current applicable black and grey sources, prioritizing reviews and authoritative guidelines where this level of evidence was available. Three key constructs were identified from the data sources as vital to the AT collaboration tool: AT stakeholder roles, AT service delivery steps and competence.

A consensus on constructs to be included within the AT collaboration tool was determined based on (i) each health practitioners understanding of best practice principles in person-focused AT service delivery [22], (ii) utilization of relevant existing tools and data sources identified during the literature review and (iii) participants expertise and lived experience knowledge.

### 2.3. Step 3: Develop AT Collaboration Tool

Data fields were then populated within key constructs using the seven AT service delivery steps (Initiate, Evaluate, Trial, Select, Procure, Implement, Review) based on each participants years of experience working in their field of work. These key constructs were discussed amongst participants to ensure consensus.

### 2.4. Step 4: Pilot AT Collaboration Tool

Each AHP was asked to independently test the AT collaboration tool developed to a common practice scenario within their discipline.

### 2.5. Step 5: Review and Revise AT Collaboration Tool

Once populated, each panel participant was to review all of the developed examples independently, then review and revise the tool collaboratively. Review and revision of the AT collaboration tool to be repeated until consensus of key constructs and data fields achieved.

## 3. Results

Four AHP participants volunteered to be involved in the tool development, piloting and subsequently authorship of this paper. Table 1 demonstrates the array of multidisciplinary perspectives across job roles, clinical streams and practice settings as well as experience as AT service users (consumers). Participants gained this experience across Australian states and territories and international workplaces and collaborations.

The panel conducted 6 months of regular virtual meetings across 3 states (VIC, SA, NSW) between September 2021—March 2022 for the final iteration.

### 3.1. Data Sources

A collaborative approach between members of the research team sought the literature upon a range of conceptual and international frameworks to inform the co-design of AT interventions. These included (i) competency standards for AHP, (ii) AT service delivery standards, and (iii) relevant Australian regulations and literature pertaining to risk management, AT education/training, and quality measures relevant to disability, aged care and AT [31,32].

#### 3.1.1. AT Stakeholders

An ecosystem of factors influences the effectiveness of AT, including AT personnel (a term which encompasses AHPs) [33]. Work is underway globally to further understand the AT ecosystem including a World Health Organization supported position paper on AT provision [22]; position paper on the roles of AT users and families [34]; and a position paper on skilled personnel [35].

In Australia, collaboration across professions already addresses complex problems in remote and rural settings [36], and primary health [37] with innovations such as transdisciplinary teams. Such initiatives represent models to safely and effectively share expertise, demonstrate a focus on shared competence and ensure practitioner governance. Collaborating with users of AHP services was less common. The literature suggests that AT stakeholders in Australia may include the AT user or consumer, their family member or circle of support; a range of AHPs and allied health assistants; and ancillary or support workforces including support workers/aged care workers, indigenous and/or disability liaison officers.

Key construct 1 for the AT collaboration tool was therefore determined to be a base set of AT stakeholder roles. The data fields within this construct were identified as:

- AT user and support network;
- disability support worker/peer supporter;
- allied health practitioner;
- team (multidisciplinary or interdisciplinary).

#### 3.1.2. AT Service Delivery Steps

**Figure 2:**
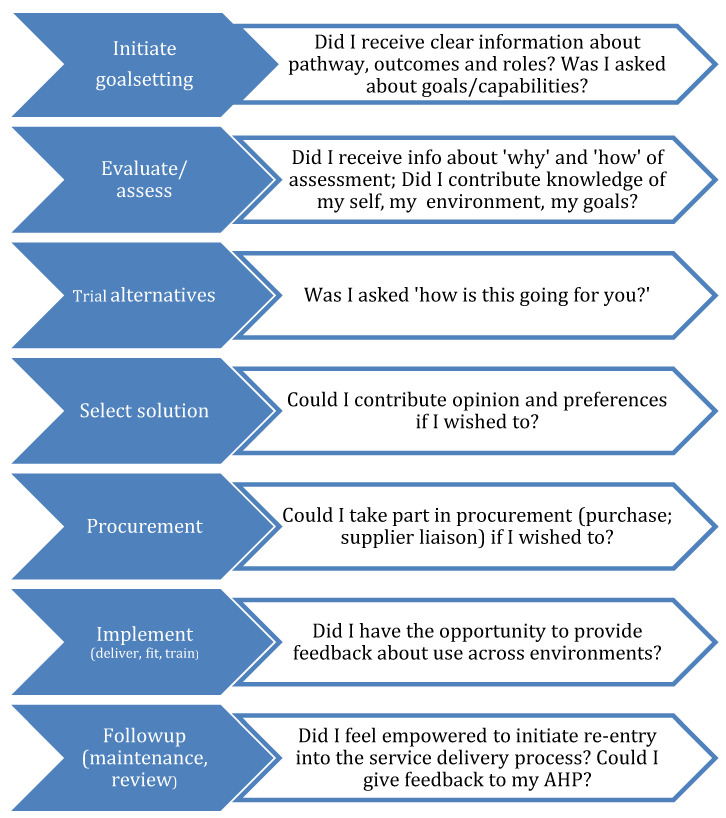
What AT users want from AT service delivery (adapted from de Jonge et al.) [38,39,40].

AT service provision steps were identified from the literature [22,24,33]. We additionally sought a consumer-empowerment approach that would foreground the desires and rights of consumers regarding their AT. Little is published by AT users themselves, hence we selected work undertaken by de Jonge et al. [38,39,40] involving consensus statements from Australian AT users regarding what they wanted from AT service delivery (Figure 2).

This series of service delivery steps was utilized within the AT collaboration tool as construct 2: AT service delivery steps. The related questions are used as a verbatim guide in the ‘consumer’ column, and as a guide within other stakeholder columns to deliver on this set of consumer priorities.

#### 3.1.3. Competence

Finding ways to role-share and collaboratively achieve outcomes clearly resonates with the ethical imperative of AHPs to empower service users: but how to do it? Competence refers to the ability to successfully perform a task, usually requiring a set of relevant knowledge and skills. A person’s capability to conduct a task in a competent way is incremental: AHPs learn these skills in tertiary training and through practitioner practice. A range of alternative workers may also take on ‘AT personnel’ roles within and beyond AHPs, for example allied health assistants [41]. International contexts present very different pictures depending upon the availability of skilled personnel, for example use of community-based rehabilitation workers in low- and middle-income countries [42]. In some jurisdictions, agreed delegations of authority combined with favorable regulatory settings have supported devolution of non-complex AT tasks. Examples of these, including evidence of their successful implementation with low cost, low risk AT include the UK Trusted Assessor Framework supported by the UK Colleges of Occupational Therapy and Physiotherapy along with several Disabled Persons Organisations [43], and Ireland’s AT Passport [44].

Given the substantial resetting of ‘prescribing’ requirements in disability (NDIS) and ageing policy (MAC) in Australia, the evidence base supporting changed policies is surprisingly scant. Some recent initiatives were identified which enable AT prescribing roles ‘beyond’ AHPs to include AT suppliers, and AT consumers as peers and mentors (ATPM).

Government funded initiatives include the 2013 NDIS funded options paper which explored national credentialing and accreditation for AT practitioners and suppliers [45]; however, its recommendations were not implemented. Additionally, in 2014, the National Disability Insurance Agency developed but did not trial an AT capability framework which scaffolds the capability of AT users according to health literacy, AT experience, length of lived experience, and the risks, complexity, and novelty of the AT in use [46,47]. Related initiatives in aged care policy include the Department of Health’s report on roles for the aged care assessment workforce in relation to AHPs [48] and funding guidelines for AT users, families and home care workers delineating AT into low risk; under advice; or prescribed, with the latter categories subject to AHP input [49].

There are currently few recognized training opportunities for an AT-focused workforce (including roles for peer mentors with lived experience), and role demarcation and relationship between such a workforce and AHP is not yet explicit.

**Figure 3:**
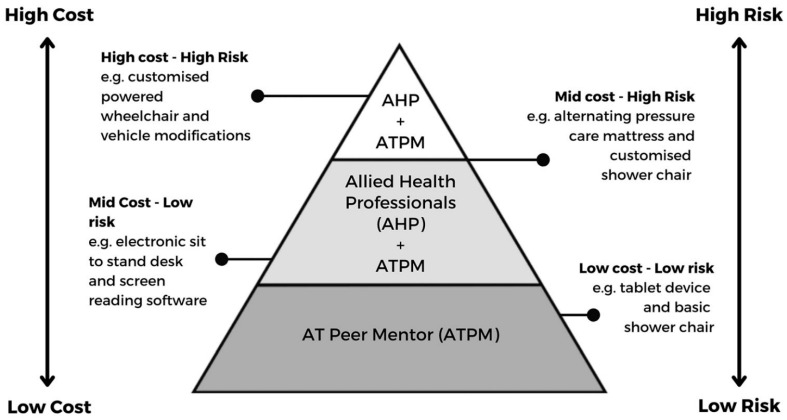
AT Risk levels and AT roles for AHP and AT Peer Mentors (ATPM) (reproduced with permission [50]).

Another model of AT peer mentorship is explicitly founded on principles of shared competence and capability between AT users and AHPs, envisioning AT users as potential AT peer mentors able to contribute to AT service delivery [50]. The AT Chat approach comprises a co-designed framework, associated training package and community of practice inclusive of AT users and AHPs. Grounded in an evidence review, the project has documented a risk-informed, competency-based and capability-enabled approach to sharing AT knowledge and roles. Features include the translation of what AT users saw as professionalized language of the AT service delivery steps into plain or ‘fit for purpose’ language, and the building of a hierarchy of AT risk levels and AT roles (See Figure 3—reproduced with permission from page 6).

Evaluation of the AT Chat pilot training and model has demonstrated that consumer’s individual capabilities can be built over time and in different ways, within relevant risk-informed quality safeguards. However different consumers want different levels of support and electing to ‘take on’ aspects of competence is in itself a choice. Such frameworks enable consumers and others who have not historically been formal actors within AT interventions, to develop capability and competency if they so choose [12].

This evidence provides a foundation for the assumption within the tool for co-design of AT interventions, that consumers may value enhanced roles, that capability will change over time and that a risk-informed, quality managed ‘sharing’ of competence is a sound strategy. For the AT collaboration tool, this work also leads us to propose a set of ‘quality indicators’, noting broad credentialing frameworks do not yet exist in Australia [45]. Key construct 3 was therefore determined to be competence in the AT intervention with data fields being any relevant quality measures which may support competence for each stakeholder, where these exist.

Based upon the above data sources, the AT collaboration tool was developed and presented in Table 2. Key construct 1: AT stakeholder roles and the data fields are listed across Row 1. These may differ depending on the AT intervention but at a minimum include the AT user, their support network and an AHP. Key construct 2: AT service delivery steps are within Column 1 with the question-based prompting data fields for this construct populated within each column under the relevant AT stakeholder roles. Key construct 3: competence in the AT intervention, and any relevant quality measures which may support competence for each stakeholder, where these exist (data fields) are populated across the bottom row again as relevant to the stakeholder role.

The tool provides a way to consider the intersections between the AT service steps, stakeholder roles, and individual competence. Questions in the tool data fields act as prompts for each stakeholder to ask about their ‘inclusion practices’, that is, have they asked others for input, and have they provided input.

To use the AT collaboration tool, AHPs and the stakeholders with whom they are working in various circumstances populate the table.

The next step entailed piloting the AT collaboration tool. Each AHP selected an exemplar scenario related to their practitioner practice to test the tool: dietetics and home enteral nutrition; prosthetics and custom prostheses; occupational therapy and vehicle modifications, and speech pathology and communication device use. Authors independently tested the AT collaboration tool by applying it to the practice scenarios, taking approximately 20 min on average (see Appendix B, Appendix C, Appendix D, Appendix E). 

Participants then shared their pilot results with the broader group. Each participant reviewed all 4 pilot results independently. Inconsistencies were identified in the way the tool was interpreted and utilized across the multiple professions represented.

A final step was review and revision of the AT Collaboration tool. Group discussion of the initial results demonstrated the constructs were broadly applicable for all four professions. The tool and its constructs and data fields were revised based on discussion and feedback to address limitations and inconsistencies and to ensure applicability across allied health professions and alternative workforces. It was noted that different interventions required different AT stakeholder role data fields, and the decision was made to enable the framework content to be adapted to be fit for purpose for specific interventions, for example, defining ‘vehicle modification agents’ (Appendix E) where 3 stakeholder roles are identified, or ‘prosthetic technicians’ (Appendix D) where 5 stakeholder roles are identified. These changes reflect the varying alternative workforces available for different AT types. This flexibility rendered the framework more specifically relevant to the breadth of scenarios AT consumers and AHPs encounter, while maintaining fidelity to the key construct of AT stakeholder roles. There were no disagreements with regards to roles and scope in reviewing each-others results.

It became apparent through this process that quality measures were both critical, and largely absent, for consumers and the support workforce, and arguably patchy for AHPs. In the absence of micro-credentials, a range of opportunities to build competence were included, and their inclusion is targeted at reminding users of the importance of assuring quality and competence as well as possibility within these constraints.

The authors as a group discussed the language and phrasing of the framework and arrived at consensus on phrasing of data items.

Participants then revised their examples independently to reflect the revised tool. This process was repeated until consensus regarding the tool constructs and data fields was achieved. Two cycles of tool iteration to arrive at a consensus regarding the key constructs and data fields for the tool which produced consistent results was required.

## 4. Discussion

To work and flourish in super-complex environments [1], AHPs must possess qualities such as, agility, recognition of personal and practitioner limitations, the ability to reflect, work collaboratively and commit to life-long learning [51]. An ethical foundation of collective allied health professions is that of consumer-centered practice [15]. Responding to the shifting policy and practice landscape led us to critically reflect on how existing systems may have supported practitioner centric models. Health and technology innovations and the rollout of consumer-directed care gives allied heath an opportunity to revisit the locus of the consumer as the center of our work. In responding to this challenge, we suggest AHPs prioritize our roles in empowering and informing other stakeholders to become competent in elements of practice. The AT collaboration tool reconceptualizes previously accepted practices from a delegation of authority approach. Initial testing of this framework suggests it may enable AHPs, and other stakeholders, to acknowledge their personal scope; may acknowledge roles for interdisciplinary teams; and articulate competency-based inputs from a range of stakeholders. It is likely when used with different AHPs that additional or alternate stakeholder categories may need to be utilized. Future testing should be focused on applicability across the remaining allied health professions for which AT tailoring is within scope.

Enacting the implementation of the tool systemically would require actions by individuals, practitioner bodies, and policymakers. Individual AHPs may take the opportunity to revisit their practices and embed roles for relevant stakeholders who would welcome enhanced engagement and capacity building, adjusting over time as individual competence levels grow and alternative workforces develop. Allied health professions, individually or collectively, may utilize models such as this to operationalize a person-centered, capacity-building approach to interventions such as AT. For systematic uptake, related aspects would need attention, such as AHP training, articulating stakeholder capacity building in competency standards, and outreach to the cohorts of AHP working in Australia. There are consequences of such a framework for policymakers and AT funders. Collaboration would be needed to discuss and embed the recognition of diverse stakeholder roles into program guidelines, risk and quality frameworks, and also to further establish quality indicators and training options. Finally, we suggest careful monitoring of outcomes to ensure safety and quality of services. Given the identified lack of structured methods for determining task competence prior to assigning roles to various stakeholders, the ever-changing competence of stakeholders and the rapid rate of new AT availability, all stakeholders will need to utilize valid and reliable population appropriate outcome measures to evaluate the suitability of outcomes. As this process is in its infancy, ongoing collaborative efforts from all parties is required to ensure consumer needs are met, and frameworks are consolidated.

## 5. Limitations and Future Research

The perspectives of four AHPs are necessarily limited, and the framework presented above, while grounded in the literature and practice knowledge, has only been piloted by the authors. In addition, these four AHPs do not comprise all allied health professions working with AT nor indeed have representation as support networks, alternative workforce or AT consumers other than some intersectional capabilities from within our group.

This paper is a starting point for future research and discussion, including the intersect of responsibility with practitioner indemnity, and further exploration of the sliding scale of competence. We also note that methods to assure quality, including credentialing remain key questions to be further explored.

At this time the AT collaboration tool is presented to facilitate further practical application by AHPs, policymakers and consumers. Given the broad nature in which competence has needed to be addressed at this time, it is considered this limitation will need to be addressed as relevant quality measures are confirmed and required by policy makers. Consistent patterns of successful and unsuccessful outcomes resulting from the use of specific quality measures included by users of the tool would assist with providing specific data fields to the competence key construct and would be welcomed. At this time, the identification of such measures are beyond the scope of the paper and the tool, however, the tool is designed in such a way that these can easily be included as it is applied to various types of AT and practice settings.

## 6. Conclusions

This paper represents a novel approach from four AHPs to apply innovative systems thinking and a consumer empowerment lens to our practice in relation to AT. Our method entailed an iterative process of critical reflections, review and revision of an AT collaboration by a panel of expert practitioner researchers. We sought a strategy to decouple the narrative of practitioner ‘territorialism’ and dominance, whilst still recognizing practitioner expertise, within the re-imagined AT policies of NDIS and My Aged Care. The AT collaboration tool is offered in recognition of the pressing need for practice and policy alignment in Australia. We suggest consumer directed care, as it is conceptualized today, requires the reconfiguring of practitioner boundaries in the interests of improved outcomes and authentic co-design with consumers as the experts of their own experiences and needs. Rethinking current practice, as typified by the AT collaboration tool, will enable AHPs to utilize their skillsets in ways that are person-focused, that utilize and support relevant networks of stakeholders, and that allow for both capability building and risk management as workforce dynamics continue to evolve.

## Figures and Tables

**Table 1:** Participant experience.

Profession	Clinical Experience	Practice Setting	Role	Years
Occupational Therapist	Health Disability Ageing	Public/Private/Non-profit Practitioner association/regulator Academic research centre Tertiary education/vocational education Australia/International	Clinical Managerial Policy Research Support roles	35
Prosthetist/ Orthotist	Health Disability Ageing	Public/Private Australia/International Practitioner association/regulator	Clinical Managerial Policy Research AT user/Consumer	21
Speech Pathologist	Health Disability Ageing	Public/Private/Non-profit Practitioner association/regulator Australia/International	Clinical Managerial Policy Research Support roles AT user/Consumer	19
Dietitian	Health	Public Academic research centre Tertiary education/vocational education Australia/International	Clinical Managerial Policy Research Education	23

**Table 2:** AT collaboration tool.

AT Stakeholder Roles				
	AT User and Support Network	Disability Support Worker/Peer Supporter	Allied Health Practitioner	Team
**AT Service Delivery Step**				
Initiate Evaluate Trial Select Procure Implement Review	Did I share my preferred role? Did I contribute my expertise about my own condition? Did I contribute knowledge of my self, my environment, my goals? Did I contribute opinion and preferences? Did I take part in procurement (purchase; supplier liaison) if I wished to? Did I have the opportunity to provide feedback about use across environments? Did I feel empowered to initiate re-entry into the service delivery process? Was I invited to give feedback to allied health practitioners?	Do I understand the person’s goals (have I asked?) Have I established my role? Did I find out what support the person would like from me? Did I take opportunities to observe and report what I know, to support the person? Did I take up opportunities to assist in skill development? Have I contributed environments for great AT uptake? What is my role in relation to maintenance & monitoring? Am I confident of my scope and to carry out my tasks? Do I need education/support/training from the AHP?	Did I ask everyone about needs and goals? Have I negotiated preferred roles with others? Have I evaluated context and environment, by asking the experts? Have I discussed AT options in an accessible way? Have I organised and evaluated adequate trial of the AT? Did I supervise and support the support network and support person’s voice? Did I ensure the person is safe and able to optimize use of their AT in all environments? Did I seek feedback regarding the quality of service? Did I seek supervision when needed? Did I refer to a specialty team if needed?	Do we understand the persons goals? Have we listened to the person and support network? Have we provided assessment and tertiary consultation? Have we enabled shared communication across the AT users support network?
Quality measures for each stakeholder				
	e.g., formal/informal advocacy training; other training	e.g., appropriate workplace credential	e.g., recognised academic training; evidence-based practice, continuing practitioner development, practicing within personal and practitioner scope	e.g., clinical practice, outcome measures guideline adherence

## Appendix A. Assistive Products Level 1 Categories from AS/ISO:9999: 2018 and AHPs

**AS/ISO 9999 Classes of Assistive Products [21]**	**AHPs Scope ***
04 Assistive products for measuring, supporting, training or replacing body functions (examples: respiratory and circulatory support products; medication management systems; feeding pumps, accessories for parenteral (intravenous) feeding)	- Dietitians - Occupational therapists - Prosthetists/Orthotists - Physiotherapists - Speech pathologists
05 Assistive products for education and for training in skills (examples: Assistive products for training alternative communication techniques and vocabulary to allow interpersonal communication; continence training products; memory training products)	- Occupational therapists - Speech pathologists
06 Assistive products attached to the body for supporting neuromusculoskeletal or movement related functions (orthoses) and replacing anatomical structures (prostheses) (examples: supportive footwear; upper limb splints, lower limb prosthesis)	- Occupational therapists - Prosthetists/Orthotists - Physiotherapists - Podiatrists - Pedorthists
09 Assistive products for self-care activities and participation in self-care (examples: adapted clothing; shower stool; belts and harnesses; positioning supports; products for tracheostomy care; continence products)	- Occupational therapists
12 Assistive products for activities and participation relating to personal mobility and transportation (examples: walking aids; wheelchairs; vehicle adaptations; transfer supports; mobile hoists)	- Occupational therapists - Physiotherapists - Exercise physiologists
15 Assistive products for domestic activities and participation in domestic life (examples: adapted cutting board; one-handed chopping equipment; wheeled laundry basket; long handled gardening equipment)	- Occupational therapists - Dietitians
18 Furnishings, fixtures and other assistive products for supporting activities in indoor and outdoor human-made environments (examples rise/recline lounge chair; adjustable bed; fold down grabrails; portable ramps)	- Occupational therapists
22 Assistive products for communication and information management (examples: assistive products for voice production; hearing loops; electronic communication devices; adapted computer mouse)	- Occupational therapists - Speech pathologists
24 Assistive products for controlling, carrying, moving and handling objects and devices (examples: environmental control units for operating devices from a distance; assistive products for extended reach; forearm support for computer use; robotic manipulator; non-slip products)	- Occupational therapists - Physiotherapists
27 Assistive products for controlling, adapting or measuring elements of physical environments (examples: air filters; products for reducing vibration; lighting management products; noise cancelling headphones)	- Occupational therapists - Physiotherapists - Dietitians - Audiologists
28 Assistive products for work activities and participation in employment (examples: workstations; lifting platforms; adapted tools; occupational health and safety products such as alarms and monitors)	- Occupational therapists - Physiotherapists - Exercise physiologists - Rehabilitation counsellors
30 Assistive products for recreation and leisure (examples: adapted saddles; sports wheelchairs; one handed playing card holders)	- Occupational therapists - Physiotherapists - Exercise physiologists
* Focussing on AHPs Who Recommend Assistive Products.	

## Appendix B. Dietetics: Co-Design in Home Enteral Nutrition (HEN)

**Person/Circle** **of Support ^1^**	**Disability Support Worker ^2^**	**Nurse or Accredited Practising Dietitian (APD)**	**Accredited Practising Dietitian (APD)**	**Multidisciplinary Team**
Deliver feed by pump, gravity or syringe	Deliver feed by pump, gravity or syringe	Care of enteral feeding tube and tube replacement depending on type of tube and skill level	Assessing and continued monitoring of nutritional requirements	
Basic trouble shooting	Basic trouble shooting	Monitor for complications related to the tube or feeding	Nutrition goals determined in collaboration with participant and evidence base eg nutrient reference values (NRV)	Assessment of ability to take oral diet and need for enteral nutrition
Basic care of enteral feeding tubes/devices and gastrostomy site	Basic care of enteral feeding tubes/devices and gastrostomy site	Training and support for patients, carer and/or disability support workers to ensure adequate skill level to provide tube care and feeding	Recommending formula, route of administration and regimen.	Insertion of enteral tube, tube changes (depending on type of tube) and management of complications
Know when to call for additional help	Know when to call for additional help		Trouble shooting	
Source information on accessing HEN products (selection of pump, delivery method)			Recommend balance between oral intake and enteral nutrition to meet nutrition requirements and agreed nutrition goals	
Active participant in decisions around HEN management			Ongoing monitoring and evaluation against nutrition goals (this includes support to achieve goals), and monitoring for nutrition-related complications	Access to specialist services to address tube and feeding complications as required
			Provision of information /prescription for accessing HEN products (feeds, pumps, giving sets, delivery method). Ensuring patient has a valid registration to order feeds and equipment. Liaising with patient, carer, disability support worker and nursing for any changes to feeding regimen	
Quality measures for each stakeholder:				
Up-to-date and ongoing AT training. Can clearly outline who to contact when assistance is needed.	Appropriate qualification. Up-to-date and ongoing training Operating within scope of practice. Regular monitoring/audit of complication rates.	Appropriate qualification. Up-to-date workplace training/credentialing/APD credentialing. Regular monitoring/audit of complication rates.	APD credential. Participant satisfaction. Ensure client is achieving their nutrition goal. Follows Regulatory body code of conduct. APD operating within person centred model.	All members appropriate qualification & trained. Follows Regulatory body code of conduct. Regular case conferences. Operating within scope of practice.
	^1^ (i.e., NDIS participant with training. Currently being trained by APD and nurse; ^2^ Appropriately qualified (Certificate IV or III) with nutrition modules (ideally with a unit on HEN care which is not currently available). Current practice is that an APD with HEN experience and/or nurse train patients/carers/support workers.			

## Appendix C. Speech Pathology: Co-Design with Communication Devices

**Person/Circle of Support**	**Disability Support Worker**	**AT Supplier/AT Mentor**	**Speech Pathologist**	**Multidisciplinary Team**
Identify need and preferences based on previous experiences	Follow communication plan developed by participant and speech pathologist	Organise and monitor adequate trial of the device/s, including training and programming of device as required	Complete thorough communication assessment across environments	Provide training to participant/support network and speech pathologist to position and access device appropriately as needed
Set goals for communication such as what a device might be used for	Basic troubleshooting	Assist with funding application as required	Assessment and tertiary consultation with other AHPs needed such as occupational therapist or physiotherapist for positioning and device access if appropriate	Receive training in using the device with the participant from speech pathologist or participant/support network depending on preference and/or skill level of participant/support network
Provide vocabulary list or communication dictionary				
Learn about the device or system and how to show others how to use it				
Basic programming and troubleshooting, depending on skill level and desire	Know when to contact others for repairs or support	Advanced training and troubleshooting support	Analysis of short- and long-term communication needs and factors leading to likely change in needs, including multi-modal approach	Use device to make sure participant is able to participate effectively in all environments
Provide feedback about what is working and what isn’t		Experience of what has worked for a range of other AT users	Programming and troubleshooting depending on skill level	
Quality measures for each stakeholder:				
Provides feedback to therapist about satisfaction with device AT assists goal achievement Remains informed of options and use in manner decided on by participant Contacts speech pathologist/team members when assistance needed	Received training Regular updates to training Proactively seeks review, maintenance and adjustments Participates in informed discussions with all other stakeholders Uses AT as intended and appropriate Documents progress as appropriate Knows who to contact when assistance needed	Appropriate qualification Received training Regular updates to training Manufacturing standards and warranty requirements met and maintained AT quality checks prior to provision	Certified practising speech pathologist status Regular supervision Uses outcome measures to ensure person has met goals Follows Regulatory body code of conduct Operating within scope of practice	Appropriate qualification Received training Regular updates to training Manufacturing Follows Regulatory body code of conduct Regular case conferences Operating within scope of practice

## Appendix D. Prosthetics and Orthotics: Co-Design with a Custom Prosthesis

**Person/Circle of Support**	**Allied Health Assistant**	**Prosthetist**	**Technician/Manufacturer**	**Multidisciplinary (MD) Team**
Set goals and share knowledge of environments of use	Assessment using relevant valid and reliable outcome measures as directed by the prosthetist and/or MD team to inform baseline assessment and progress toward goal achievement	Assessment of ability to use prosthesis in line with goals Determine goals in collaboration with participant based on outcome measure and assessment findings	Manufactures prosthesis as directed by Prosthetist according to applicable standards and instructions	Physiotherapist and/or Occupational Therapist provide education and training in use of AT, particularly within specific environments as well as care of whole self when using
Maintain AT	Provides person with and receives from others ongoing education and training in use as directed by prosthetist and/or MD team	Discuss advantages and disadvantages of all prosthesis options in line with evidence base	Sources required parts to be included in prosthesis	Assessment, discussion and provision as required of complementary AT
Use AT safely and within warranty requirements		Shared decision making regarding type of prosthesis to be used		Nursing/Podiatrist-wound management as required
Troubleshoot fit and use challenges independently and seek assistance as appropriate		Assists person to access financial support for AT and related services as required		
Trial where possible and report subjective and objective feedback Research available options to enable informed discussion and choice regarding prosthetic parts		Ensure optimal fit, function and safety of prosthesis in an ongoing manner Provide education re use, care and maintenance requirements of AT in collaboration with MD Team, Allied Health Assistant and persons support network		Medical-access to specialist services and/or medications to enable use as required e.g. pain medication, mental health assistance, investigative measures
Informed of and able to share information related to payment options & preferences				
Quality measures for each stakeholder:				
Received training Regular updates to training Proactively remains informed of options and use Proactively seeks review, maintenance and adjustments Participates in informed discussions with all other stakeholders as preferred Uses AT as intended and appropriate AT assists goal achievement	Appropriate qualification Regular updates to training Operates within scope of practice Operates within person centred model	Appropriate qualification Operates within scope of practice Participates in continued learning, provides and receives supervision Remains aware of all AT options Integrates current evidence to practice Operates within person centred model Seeks feedback from all stakeholders Uses outcome measures to ensure person has met goals	Appropriate training and supervision Manufacturing standards and warranty requirements met and maintained AT quality checks prior to provision	Appropriate qualification Participates in continued learning, remains aware of all AT options, integrates current evidence to practice Operates within person centred model Operates within scope of practice Uses outcome measures to ensure person has met goals

## Appendix E. Occupational Therapy and Co-Design in Vehicle Modifications

**Person/Circle of Support**	**AT Supplier/Vehicle Modification Agent**	**Occupational Therapist**
Goal identification and knowledge of environments of use		Goalsetting and establish outcomes
Knowledge of transfer capacity		Assess participant [physical capacity; future needs; balance; reach range; endurance; cognition]
Understanding of physical and organisational capacity		Assess environments of use [parking and driving clearances and distances; path of travel; transfer and handling implications of trailers, hoists and ramps]
Source information on vehicle modification options	Knowledge regarding product options and vehicle ‘fit’ with range of products	
Envision the link between all these factors, when looking at available options on the market		
Determine when to consult experts to determine the solution which will fit person, task and environment	Experience of what has worked for a range of other AT users	Task analysis [physical and cognitive demands of transfers to carseat; ambulating to ramp/trailer; handling of hardware]
Quality measures for each stakeholder:		
Develop own experience-based knowledge of needs and potential solutions Learn how to utilise evidence based resources such as National Equipment Database Skilled in appraising products as a consumer ie familiar with pro and con evaluation; utilise consumer and fair trading resources.	No formal training/credentialing exists Maintains currency with market and modification technology	No formal training/credentialing exists May complete driver-related certificate course which partially covers competencies Participate in community of practice for vehicle modifications Engages in appropriate mentoring/supervision relationships to maintain currency of skill
